# Association between Total and Individual PCB Congener Levels in Maternal Serum and Birth Weight of Newborns: Results from the Chiba Study of Mother and Child Health Using Weighted Quantile Sum Regression

**DOI:** 10.3390/ijerph19020694

**Published:** 2022-01-08

**Authors:** Akifumi Eguchi, Kenichi Sakurai, Midori Yamamoto, Masahiro Watanabe, Aya Hisada, Tomoko Takahashi, Emiko Todaka, Chisato Mori

**Affiliations:** 1 Division of Environmental Preventive Medical Sciences, Center for Preventive Medical Sciences, Chiba University, Chiba 263-8522, Japan; sakuraik@faculty.chiba-u.jp (K.S.); midoriy@faculty.chiba-u.jp (M.Y.); m_watanabe@chiba-u.jp (M.W.); a_hisada@chiba-u.jp (A.H.); todakae@faculty.chiba-u.jp (E.T.); cmori@faculty.chiba-u.jp (C.M.); 2 Department of Environmental Preventive Medicine (Yamada Bee Company, Inc.), Center for Preventive Medical Sciences, Chiba University, Chiba 263-8522, Japan; t.takahashi@chiba-u.jp; 3 Department of Bioenvironmental Medicine, Graduate School of Medicine, Chiba University, Chiba 260-8670, Japan

**Keywords:** polychlorinated biphenyls, congeners, birth weight, prenatal exposure

## Abstract

Maternal exposure to polychlorinated biphenyls (PCBs) during pregnancy is associated with a low birth weight; however, the congener-specific effects of PCB congeners are not well defined. In this study, we used maternal serum samples from the Chiba Study of Mother and Child Health (C-MACH) cohort, collected at 32 weeks of gestational age, to analyze the effects of PCB congener exposure on birth weight by examining the relationship between newborn birth weight and individual PCB congener levels in maternal serum (n = 291). The median total PCB level in the serum of mothers of male and female newborns at approximately 32 weeks of gestation was 39 and 37 ng g^−1^ lipid wt, respectively. The effect of the total PCB levels and the effects of PCB congener mixtures were analyzed using a linear regression model and a generalized weighted quantile sum regression model (gWQS). The birth weight of newborns was significantly associated with maternal exposure to PCB mixtures in the gWQS model. The results suggest that exposure to PCB mixtures results in low newborn birth weight. However, specific impacts of individual PCB congeners could not be related to newborn birth weight.

## 1. Introduction

Low newborn birth weight is associated with the development of various diseases, such as hypertension in later life [1,2,3,4]. The “Developmental Origins of Health and Disease” (DOHaD) hypothesis also states that environmental factors may adversely affect the development of the fetus, leading to several lifestyle-related diseases, such as obesity, diabetes, hypertension, and mental disorders [5,6,7]. Therefore, the effects of exposure to various environmental pollutants during the fetal period warrants attention [8].

Polychlorinated biphenyls (PCBs) have not been manufactured since the 1970s because of their toxicity to human health. However, they remain in the environment and are still detected in human tissues owing to their persistent nature [9,10]. Studies indicated that PCBs are detectable in umbilical cord blood because they pass through the placenta [11,12], raising concerns that they might adversely affect the normal development of fetuses [13,14,15]. Several studies investigated the relationship between PCB exposure and low birth weight [16,17]. For example, it was reported that the exposure to mixtures of persistent endocrine-disrupting chemicals might be related to low birth weight, with a high contribution from per- and polyfluoroalkyl substances (PFAS) [18]. However, the specific effects of individual PCB congeners are not interpreted well [19], as was also the case in our previous study [16].

The Chiba Study of Mother and Child Health (C-MACH)—a birth cohort study in Japan that focused on the effects of environmental health on fetuses—has been conducted since 2014 [20] (Sakurai et al., 2016). In our study, maternal serum samples from the C-MACH cohort were collected at approximately 32 weeks of gestational age to examine the relationship between newborn birth weight and exposure to individual PCB congeners during the fetal period.

In this study, we attempted to control this bias by using the multiple imputation method to impute values below the limit of detection. Furthermore, to avoid multicollinearity effects, we analyzed the relationship between health outcome and exposure using the weighted quantile sum (WQS) regression, which is suitable for analyzing the effects of exposure to chemicals that are strongly correlated with one another [21]. One study indicated that WQS has greater sensitivity and specificity in identifying the elements associated with a health outcome than ordinary linear regression analysis and linear regression analysis with regularization under simulated conditions, where the subjects are exposed to mixtures of strongly correlated chemicals [22].

## 2. Materials and Methods

### 2.1. Sample Collection

Recruitment began in February 2014 and ended in June 2015. Consent for participation in the C-MACH was obtained from 433 women. Twenty-five women withdrew from the study after providing informed consent, resulting in a final cohort of 408 women [20]. Cohorts from three OB/GY hospitals in Chiba and Saitama prefectures (Chiba: 2, Saitama: 1) in Japan were used in this study to investigate PCB levels in maternal serum samples, facilitating the study of PCB exposure on newborn birth weight. Personal data and serum samples were available for 291 of the 408 participants (age, body mass index [BMI], drinking habits, and the number of deliveries; serum: samples at a gestational age of 32 weeks and smoking habits at a gestational age of 12 weeks), and their newborns (gestational weeks at birth, maternal weight gain, and birth weight), through questionnaires and medical records (Table 1). Whole blood samples were collected, and serum samples were obtained via centrifugation and stored at −80 ℃ for PCB analysis.

### 2.2. PCB Analysis

Serum concentrations of 17 PCB congeners (CB66, 74, 105, 118, 126/178 [co-elute], 138, 146, 153, 156, 170, 177, 180, 183, 187, 194 and 201) were analyzed using gas chromatography (GC) with negative ion chemical ionization qMS (NICI-qMS), as described by Eguchi et al. [23], because these congeners are representative ones of the PCBs, which are account of more than 50% in the total PCBs [24]. PCB analysis was performed on a JMS-Q1050GC (JEOL Ltd., Tokyo, Japan) quadrupole mass spectrometer, equipped with an Agilent 7890 B gas chromatograph and a 7693 autosampler (Agilent Technologies Inc., Tokyo, Japan). GC separation was achieved using an HP5-MSUI fused-silica capillary column (30 m × 0.25 mm ID × 0.25 μm film, Agilent Technologies Inc., Tokyo, Japan). The identification and quantification of the 16 PCB congeners were achieved by monitoring the chlorine ion [Cl^−^: m/z: 35] through selected ion monitoring (SIM) analysis using the NICI-MS detector. Our analytical protocols were accredited by the Japan Accreditation Board and compliant with ISO/IEC 17025:2005 standards. During the entire analytical sequence, blank determinations were carried out at intervals of 20 samples to identify contamination. The detection limits for individual PCBs in this study ranged from 0.2 to 0.8 ng g^−1^ fat wt, and PCB congener surrogate recovery rates ranged from 70.7 to 140%. For quality control and assurance of the PCB analysis, our laboratory participated in an inter-calibration exercise based on the Standard Reference Material 1957; PCB values determined in this study were typically within the certified range [23].

### 2.3. Statistical Analysis

Data processing and analysis were performed using R ver. 4.0.5. Correlations between exposures and outcomes were determined using Spearman’s rank correlation coefficient with the R package *corrplot* (https://cran.r-project.org/web/packages/corrplot/index.html, accessed on 5 December 2021). PCB concentrations below the limit of detection (LOD) were assigned according to LOD/√2 for Spearman’s rank correlation analysis.

Differences based on newborn sex, the hospital of birth, in exposure, covariates, and outcomes were determined using the Kruskal–Wallis test with the R package *tableone* (https://cran.r-project.org/web/packages/tableone/index.html, accessed on 5 December 2021). A linear regression model was implemented using the base R function. Linear WQS with multiple imputations was used to calculate samples with PCB concentrations below the LOD and investigate the effects of PCB mixtures using the gWQS package [25]. WQS regression is a model for estimating the combined effect of all exposure variables and covariates on a health outcome in a high-dimensional dataset [21]. The chemicals included in the index are constrained to have an effect in the same direction or no effect. The application of WQS is limited to settings where it is reasonable to combine all the chemicals into a single index. The chemical indicators for WQS are assumed to affect the health outcome in the same direction [26]. In this study, PCB isomers were selected as chemical indicators affecting health outcomes, making this assumption valid. For linear regression 100 imputed data were generated for congener values below the LOD (leftcenslognorm), weight gain during pregnancy (norm), drink habit (logreg), and smoking habit (polyreg) using mice [27] package. The “leftcenslognorm” function was built into the qgcomp [28] package. Additionally, for WQS analyses, 100 imputed data were generated, and weights for individual PCB congeners were obtained across 2000 bootstrap samples. In this study, PCB congeners detected in at least 50% of the samples were used for the statistical analysis. The type I error rate was fixed at 0.05 in all statistical analysis, and quartiles were used in WQS model.

## 3. Results

Details of the individual participants, including serum PCB levels, are listed in Table 1. Since previous studies have indicated gender differences in the exposure effects of PCBs [18], we attempted to compare gender differences for basic characteristics. In basic characteristics, birth weight was the only factor that was found to be significantly different between male and female infants. The median total PCB level in serum of mothers bearing male and female fetuses at approximately 32 weeks of gestation was 39 and 37 ng g^−1^ lipid wt, respectively. The detected PCB congeners were correlated with one another (r_s_: 0.49–0.99, Figure S1, Supplementary Materials). CB 153, 138, and 180 were the dominant PCB congeners in maternal serum, and the profiles were similar to those in previous Japanese studies on maternal serum [29,30,31]. Since the major source of exposure to the main PCB congeners detected in the serum of Japanese individuals is fish consumption (50–90%) [30], the high correlations found between each PCB congener and the PCB profiles may be a reflection of the high dietary intake of fish [32]. On the other hand, correlation coefficients between CB126-178 (CB126 and 178 were co-eluted) and the other congeners (r_s_: 0.49–0.71. Figure S1), and between CB74 and the other congeners (r_s_: 0.53–0.78. Figure S1) were slightly lower, owing to the low detection rate of these congeners, differences in the source of exposure, and the difference in the speed of metabolism of PCB which has fewer chlorine atoms on biphenyl [33].

The results of the linear regression model are presented in Table 2. In this study, the total PCB levels were not significantly associated with newborn birth weight, even when stratified based on their sex and covariates with and without weight gain during pregnancy. When all PCB congeners with detection frequencies higher than 50% were included in the multiple linear regression model with multiple imputations, no correlation was observed between birth weight and any of the PCB congeners (data not shown). Additionally, in the models considering weight gain during pregnancy, the PCB mixture was not significantly related to newborn birth weight (Table 3, WQS model 1–3). On the other hand, in the WQS model, exposure to the PCB mixture was negatively associated with newborn birth weight (β = −48.0, *p* = 0.046, Table 3, WQS model 4) in the model without weight gain during pregnancy. After stratification by sex of the newborns, even in the models were not significant, the effect of exposure on birth weight for both groups were observed to be similar to that for the unstratified model (male newborns: β = −40.2, *p* = 0.26, female newborns: β = −45.9, *p* = 0.17, Table 3, WQS model 5 and 6) in the model without weight gain during pregnancy. These results indicated that sex of the newborn does not influence the effect of exposure to PCBs on birth weight. This relationship was not evident in models that considered weight gain during pregnancy, which indicate that the sum of maternal own weight gain and the weight of a fetus with their adnexa may have obscured the effects of other variables.

There are two possible reasons for the different results obtained using the WQS and linear regression models. First, multicollinearity was avoided with correlation observed among the congeners in the mixture of PCBs in WQS, which is the unidirectionality of the index and the ensemble step; second, the WQS model can evaluate the mixture effects of PCB congeners [26]. Although the mechanism of the relationship between decreased birth weight and PCB exposure is not fully understood, previous studies have reported that disturbances in maternal hormonal homeostasis and metabolism may be related to PCB exposure [17,34].

**Figure 1:**
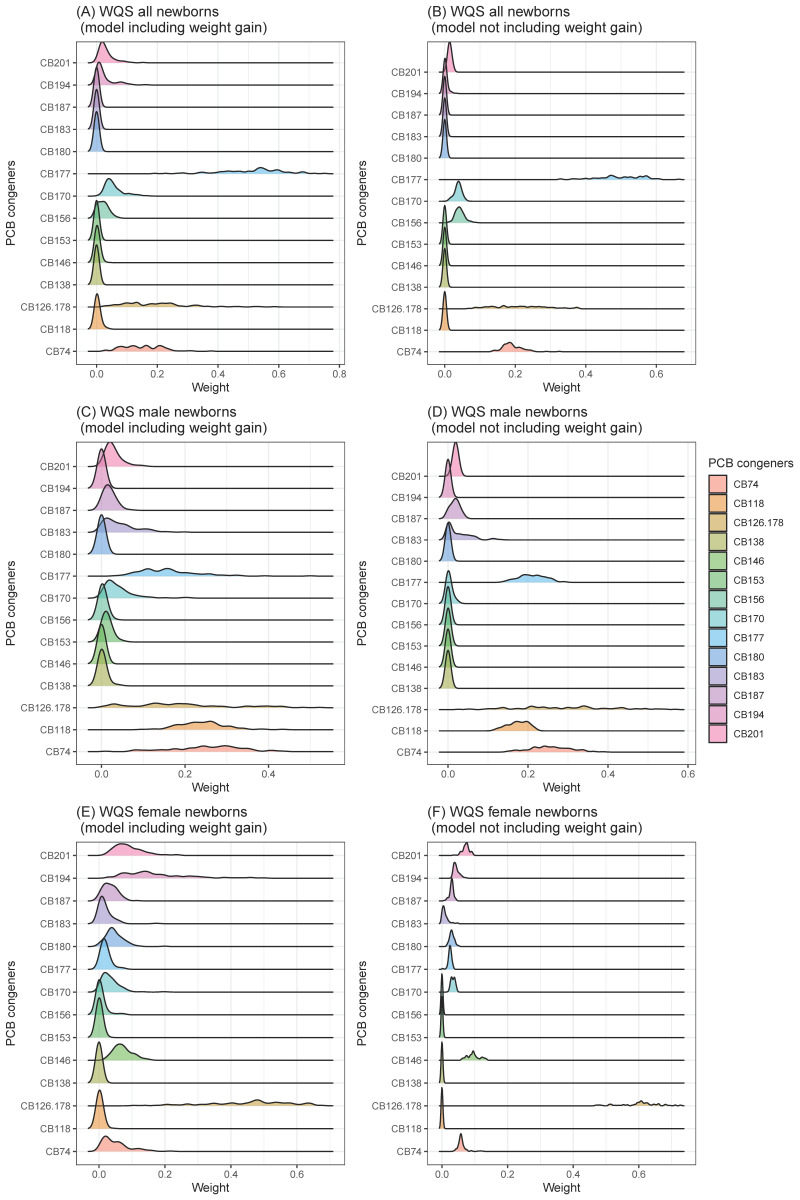
Distributions of importance weights of PCB congeners in WQS models (value range: 0–1, A larger value suggests that it is more important in the model). Distributions of importance weights were generated based on importance weights for each congener in 100 WQS models calculated based on multiple imputed data ((**A**): all newborns, model including weight gain, (**B**): all newborns, model not including weight gain, (**C**): male newborns, model including weight gain, (**D**): male newborns, model not including weight gain, (**E**): female newborns, model including weight gain, (**F**): female newborns, model not including weight gain).

Due to the specific effects of individual PCB congeners not being well determined in our previous study [16], we explored the importance of weight of the individual PCB congeners for birth weight using gWQS in this study. Focusing on the effect of individual PCB congeners in the WQS model, some PCBs had stronger influences on birth weight (all: CB74, CB177, male: CB74, 118, and 177 and female: CB 126_178) (Figure 1).

However, the importance of weights of individual PCB congeners of gWQS were not consistent, indicating that the effects of PCB exposure on birth weight may not be congener-specific (Figure 1). Since individual PCB congeners have different toxicities, and they may exert varied effects on birth weight. One study found that pre-conceptional exposure to a combination of antiestrogenic PCB congeners (CB77_110: co-elute, 105, 114, 126, 156_171: co-elute, and 169) was responsible for low birth weight, while the cumulative effect of other types of PCB congeners was not significantly related to newborn birth weight [19]. However, in our study, the impact of the detected antiestrogenic PCB congeners, determined using the WQS model, was relatively low. The reason for this difference is not known, because the total PCB concentration includes coplanar PCBs with dioxin-like structures [35]. Furthermore, several studies have suggested that changes in structure may alter the toxicity of PCBs [19]. It is important to monitor the effects of each congener in more detail using large-scale studies, which should be the focus of future work.

## 4. Conclusions

Maternal serum samples from the C-MACH cohort, collected at a gestational age of 32 weeks, were used to examine the relationship between newborn birth weight and individual PCB congener levels. Linear regression and gWQS models were used in this study, and results from the gWQS model suggested that PCB mixture exposure was associated with low newborn birth weight. It was, however, observed that the impact on birth weight from antiestrogenic PCB congeners detected in the serum was relatively low. Unlike PCB mixtures, a significant correlation was not observed between individual congeners and birth weight, indicating that the effects of PCB exposure on birth weight may not be congener-specific.

While these data provide insight into the potential association of PCB exposure and low birth weight, we acknowledge that our study has several limitations. For instance, unmonitored contaminants, including unmonitored PCB congeners in this study may have contributed to low birth weights in addition to the PCBs measured in this study. Other important limiting factors include the lack of information on PCB exposure before pregnancy, and a small sample size, which may have affected the study results.

Further research on a larger scale is required, including detailed studies on the individual effects of each congener.

## Figures and Tables

**Table 1:** Individual details of participants.

		Overall	Male	Female	*p*-Value
n	% Below Limit of Detections	291	147	144	(Male: Female)
Age (median [IQR])		33.0 [30.0, 36.0]	33.0 [29.0, 36.0]	32.0 [30.0, 36.0]	0.847
Maternal height (median [IQR])		159.0 [155.6, 162.3]	159.0 [156.0, 163.0]	159.0 [155.4, 162.0]	0.208
Maternal weight before pregnancy (median [IQR])		52.3 [48.0, 57.4]	53.0 [49.0, 57.0]	51.8 [47.0, 58.0]	0.284
Maternal BMI before pregnancy (median [IQR])		20.7 [19.2, 22.5]	20.8 [19.3, 22.6]	20.7 [19.0, 22.4]	0.751
Maternal weight gain during the pregnancy (mean (SD))		10.20 (3.36)	10.44 (3.16)	9.94 (3.56)	0.324
Parity (mean (SD))		1.66 (0.47)	1.63 (0.49)	1.69 (0.46)	0.218
Gestational weeks (median [IQR])		39 [38, 40]	39 [38, 40]	39 [38, 40]	0.426
Alcohol habit during pregnancy (Yes)		7 (2.4%)	3 (2.0%)	4 (2.8%)	0.978
Smoking habit including past (Yes)		64 (22.1%)	36 (24.7%)	28 (19.4%)	0.353
CB66 (median [IQR]) *	63.2	<LOD [<LOD, 0.35]	<LOD [<LOD, 0.37]	0.00 [<LOD, 0.34]	0.193
CB74 (median [IQR]) *	9.72	0.99 [0.68, 1.5]	1.0 [0.70, 1.5]	0.95 [0.66, 1.4]	0.193
CB105 (median [IQR]) *	50.2	<LOD [<LOD, 0.70]	<LOD [<LOD, 0.65]	0.43 [<LOD, 0.72]	0.228
CB118 (median [IQR]) *	6.25	2.2 [1.6, 3.3]	2.4 [1.50, 3.6]	2.1 [1.6, 3.2]	0.367
CB126/178 [co-elute] (median [IQR]) *	33.3	0.51 [<LOD, 0.74]	0.51 [<LOD, 0.71]	0.53 [<LOD, 0.75]	0.66
CB138 (median [IQR]) *	0	6.5 [4.8, 9.1]	7.0 [5.2, 9.2]	6.2 [4.6, 8.8]	0.088
CB146 (median [IQR]) *	4.17	1.7 [1.2, 2.2]	1.7 [1.3, 2.4]	1.7 [1.2, 2.1]	0.301
CB153 (median [IQR]) *	0	9.42 [7.11, 13.34]	9.7 [7.3, 14]	9.4 [7.0, 13]	0.201
CB156 (median [IQR]) *	9.72	1.0 [0.67, 1.4]	1.0 [0.71, 1.4]	0.99 [0.67, 1.5]	0.571
CB170 (median [IQR]) *	1.3	2.4 [1.7, 3.1]	2.6 [1.8, 3.2]	2.2 [1.7, 3.0]	0.071
CB177 (median [IQR]) *	24.3	0.80 [0.54, 1.1]	0.83 [0.55, 1.1]	0.79 [0.53, 1.0]	0.161
CB180 (median [IQR]) *	0	5.6 [4.0, 7.3]	5.6 [4.2, 7.5]	5.4 [3.7, 7.2]	0.292
CB183 (median [IQR]) *	29.2	0.63 [<LOD, 0.93]	0.64 [<LOD, 0.94]	0.63 [<LOD, 0.92]	0.919
CB187 (median [IQR]) *	0	2.7 [2.1, 3.9]	2.8 [2.2, 3.9]	2.7 [2.0, 3.7]	0.435
CB194 (median [IQR]) *	13.9	0.84 [0.63, 1.2]	0.88 [0.62, 1.2]	0.89 [0.64, 1.2]	0.527
CB201 (median [IQR]) *	12.5	0.98 [0.68, 1.2]	0.92 [0.68, 1.2]	0.94 [0.67, 1.2]	0.568
Total PCB (median [IQR]) *		37 [27, 50]	39 [28, 52]	37 [27, 48]	0.189
Birth weight of newborns (median [IQR])		3095 [2894, 3339]	3170 [2918, 3395]	3045 [2876, 3214]	0.007

* PCB levels: ng g^−1^ lipid wt.

**Table 2:** Coefficients (estimate), standard error, and *p*-values from linear regression models for all participants and their newborns.

	Estimate	SE	*p*-Value
Linear model 1: All participants *	−0.849	1.04	0.42
Linear model 2: Male newborns **	−0.182	1.28	0.89
Linear model 3: Female newborns **	−1.94	1.79	0.28
Linear model 4: All participants ***	−1.47	1.03	0.16
Linear model 5: Male newborns ****	−0.827	1.27	0.52
Linear model 6: Female newborns ****	−2.61	1.77	0.14

* Covariates: maternal age, maternal BMI, number of children, gestational weeks, sex of children, weight gain during pregnancy, drink habit, and smoking habit. Exposure: Total PCB level; ** Covariates: maternal age, maternal BMI, number of children, gestational weeks, weight gain during pregnancy, drink habit, and smoking habit. Exposure: Total PCB level; *** Covariates: maternal age, maternal BMI, number of children, gestational weeks, sex of children, drink habit, and smoking habit. Exposure: Total PCB level; **** Covariates: maternal age, maternal BMI, number of children, gestational weeks, drink habit, and smoking habit. Exposure: Total PCB level.

**Table 3:** Coefficients (estimate), standard error, and *p*-values from weighted quantile sum regression models for all participants and their newborns.

	Estimate	SE	*p*-Value
WQS model 1: All participants *	−40.6	24.3	0.095
WQS model 2: Male newborns **	−26.8	35.8	0.46
WQS model 3: Female newborns **	−46.4	35.2	0.19
WQS model 4: All participants ***	−48.0	23.9	0.046
WQS model 5: Male newborns ****	−40.2	35.4	0.26
WQS model 6: Female newborns ****	−45.9	32.9	0.17

* Covariates: maternal age, maternal BMI, number of children, gestational weeks, sex of children, weight gain during pregnancy, drink habit, and smoking habit. Exposure: Total PCB level; ** Covariates: maternal age, maternal BMI, number of children, gestational weeks, weight gain during pregnancy, drink habit, and smoking habit. Exposure: Total PCB level; *** Covariates: maternal age, maternal BMI, number of children, gestational weeks, sex of children, drink habit, and smoking habit. Exposure: Total PCB level; **** Covariates: maternal age, maternal BMI, number of children, gestational weeks, drink habit, and smoking habit. Exposure: Total PCB level.

## Data Availability

The datasets generated and analyzed during the current study are available from the corresponding author on reasonable request. The data are not publicly available due to their containing information that could compromise the privacy of research participants.

## Supplementary Materials

The following are available online at https://www.mdpi.com/article/10.3390/ijerph19020694/s1, Figure S1: Correlation matrix of PCB congeners and birth weight.
