# Supplementary figures and images for: Association between Total and Individual PCB Congener Levels in Maternal Serum and Birth Weight of Newborns: Results from the Chiba Study of Mother and Child Health Using Weighted Quantile Sum Regression
Source: Int J Environ Res Public Health. 2022 Jan 8;19(2):694. doi: 10.3390/ijerph19020694 (PMC8775854; doi:10.3390/ijerph19020694)

## Slide 1
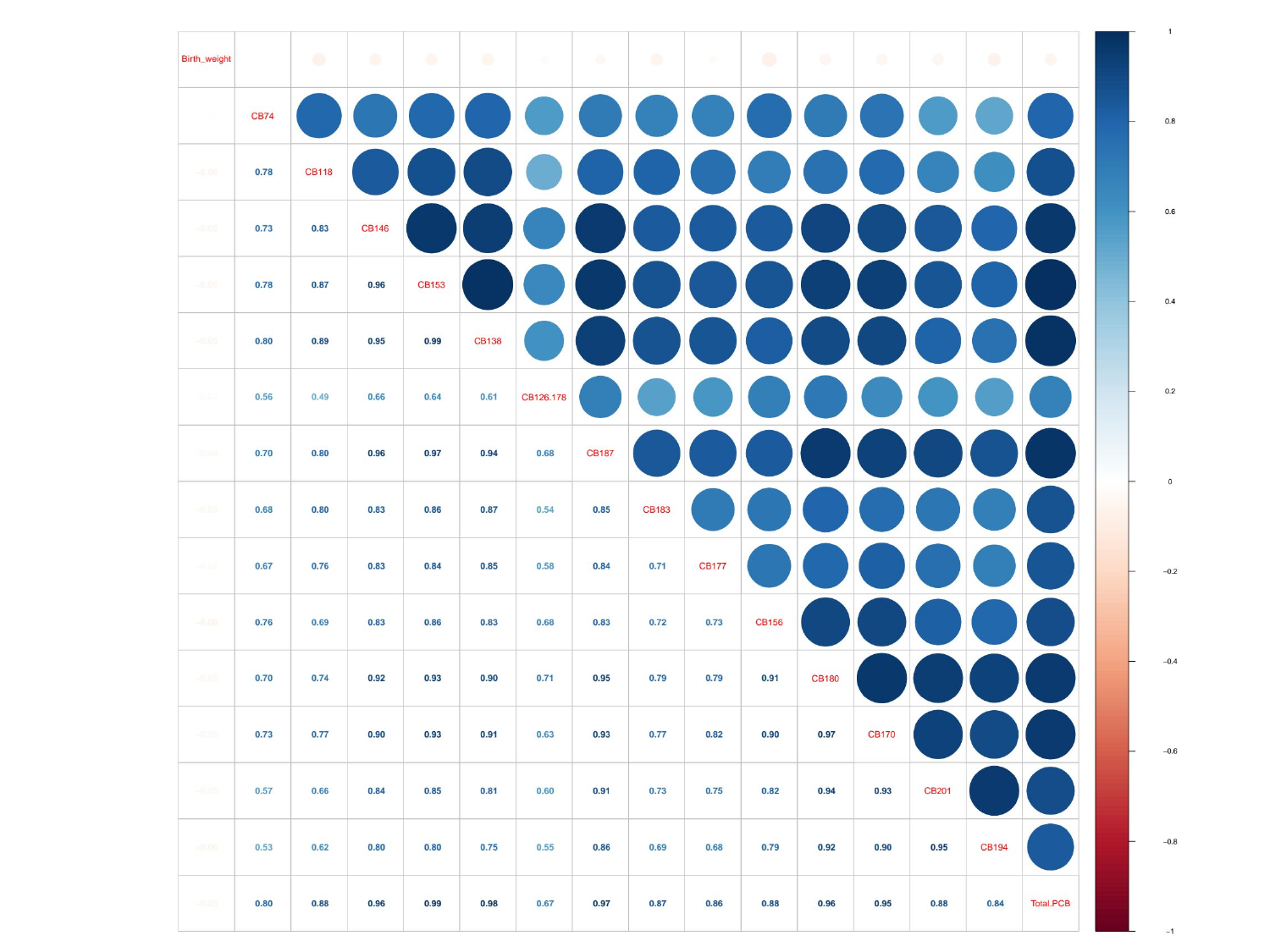

Supplement: Supplementary file 1 [file ijerph-19-00694-s001.zip › ijerph-1519427-Supplementary Figure S1.pptx]
